# Clinico‐cytopathologic analysis of 574 Pericardial Effusion Specimens: Application of the international system for reporting serous fluid cytopathology (ISRSFC) and long‐term clinical follow‐up

**DOI:** 10.1002/cam4.4408

**Published:** 2021-11-07

**Authors:** Min Jeong Song, Uiree Jo, Ji‐Seon Jeong, Kyung‐Ja Cho, Gyungyub Gong, Yong Mee Cho, Joon Seon Song

**Affiliations:** ^1^ Department of Pathology Asan Medical Center University of Ulsan College of Medicine Seoul Korea

**Keywords:** cytology, malignancy, outcomes, pericardial effusion

## Abstract

**Introduction:**

A pericardial effusion (PE) has a variable etiology and the primary role is diagnosis of metastatic malignancy. We analyzed the PE cytology in a large cohort in accordance with the international system for reporting serous fluid cytopathology (ISRSFC) and evaluated the long‐term patient outcomes.

**Methods:**

PE specimens from 2010 to 2014 with an available clinical history, cytologic data, and pericardial biopsy results were collected.

**Results:**

A total of 574 PE specimens were obtained from 486 patients, representing 1.5% (574/38,589) of all body fluid specimens. Three hundred and eighty‐two (66.6%) cases were “negative,” 54 (9.4%) cases were “atypia of undetermined significance,” 10 (1.7%) cases were “suspicious for malignancy,” and 128 (22.3%) cases were “malignancy”. The most common origin for malignant PE was the lung (82.1%), in both men (70.5%) and women (50.6%). Breast cancer (20%) in women and gastric cancer (4.9%) in men were the second most common malignant PE, respectively. The mean interval from the occurrence of malignant PE to death was 10.06 months (range; 0–116.03 months, median 3.5 months), and the 1‐year survival rate was 16.7%. In addition, the 1‐year survival rates after malignant PE onset were 0% for gastric cancer, 13.9% for lung cancer, 19.8% for breast cancer, and 21.1% for the other cancers (*p* = 0.011).

**Conclusion:**

Our present study is the first to our knowledge to classify the pericardial fluid from 574 cases in accordance with the recently published ISRSFC, and to present the long‐term outcomes of patients with malignant PE at the same time. Moreover, we report for the first time that it is gastric and not lung cancer patients that have the poorest prognosis after the occurrence of malignant PE.

## Graphical abstract

Our study report the long‐term outcomes of patients with malignant pericardial effusion. In addition, most common malignant pericardial effusion was lung cancer and gastric cancer showed the poorest survival prognosis after the occurrence of malignant pericardial effusion.

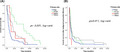

## 1 INTRODUCTION

Body fluids including pleural, peritoneal, and pericardial fluids accumulated in pathologic conditions, including benign and nonneoplastic disorders, and benign and malignant neoplasms.^1^, ^2^, ^3^ Cytologic evaluations are among the diagnostic tools used to determine the etiology of a pericardial effusion (PE), and in particular to test for a possible malignancy.^4^

The pericardium is a double‐walled sac containing the heart and roots of the great vessels and is composed of both serous and fibrous pericardium. The serous pericardium is divided into the parietal pericardium and visceral pericardium. Both of these layers lubricate against the friction that occurs during heart activity. Hence, 20 to 60 ml of fluid normally accumulates in the pericardial space.^5^ PE accumulation is caused by variable mechanisms in a similar manner to other body fluids including infection, malignancy, connective tissue disease, hemodynamic instability, and idiopathic causes.^6^, ^7^, ^8^ It results in considerable morbidity and contributes to mortality. A systemic evaluation of PE cytology is rare in the literature however compared to pleural or pericardial effusions.^9^

“The International System for Reporting Serous Fluid Cytopathology (ISRSFC)” was recently established to create a reporting system for serous fluid cytopathology, and has been endorsed by the International Academy of Cytology (IAC) and the American Society of Cytopathology (ASC).^10^ The purpose of the ISRSFC is to develop an evidence‐based diagnostic system along with management recommendations that will enhance professional communication among clinicians and other medical staff, and thus improve patient care.

Herein, we analyzed the PE cytology on a large scale in accordance with the ISRSFC and thereby analyzed the long‐term outcomes of the patients in our cohort.

## 2 MATERIALS AND METHODS

### 2.1 Patient and sample collection

This study was performed in accordance with protocols approved by Institutional Review Board of Asan Medical Center (2021‐0878). PE specimens that had been collected from January 2010 to December 2014 at our hospital were retrieved from the medical records and both the pathologic reports on these patients, and their clinical data such as age, sex, primary tumor location, treatment, outcomes, and cytologic features, were reviewed. The slides including liquid‐based cytology and cell blocks of all cases were re‐reviewed by two certified pathologists (MJS and JSS) to verify the diagnosis.

### 2.2 Pericardial effusion preparation

Fresh specimens were received and prepared according to standard clinical processing. The PE preparation method used has been described for effusions in previous reports.^11^, ^12^, ^13^ Briefly, effusions were either entirely submitted for centrifugation or a representative 15‐ml sample was processed. During processing, specimens were divided into two tubes and centrifuged. One of the tube was used for preparing two slides using the cytospin method (Thermo Fisher Scientific) and stained with the Papanicolaou stain. A cell block was routinely prepared for all samples unless there was inadequate material. A cell pellet was obtained from the other tube and the material was fixed in formalin, processed as a cell block, and stained with hematoxylin and eosin.

### 2.3 Cytologic classification

**FIGURE 1:**
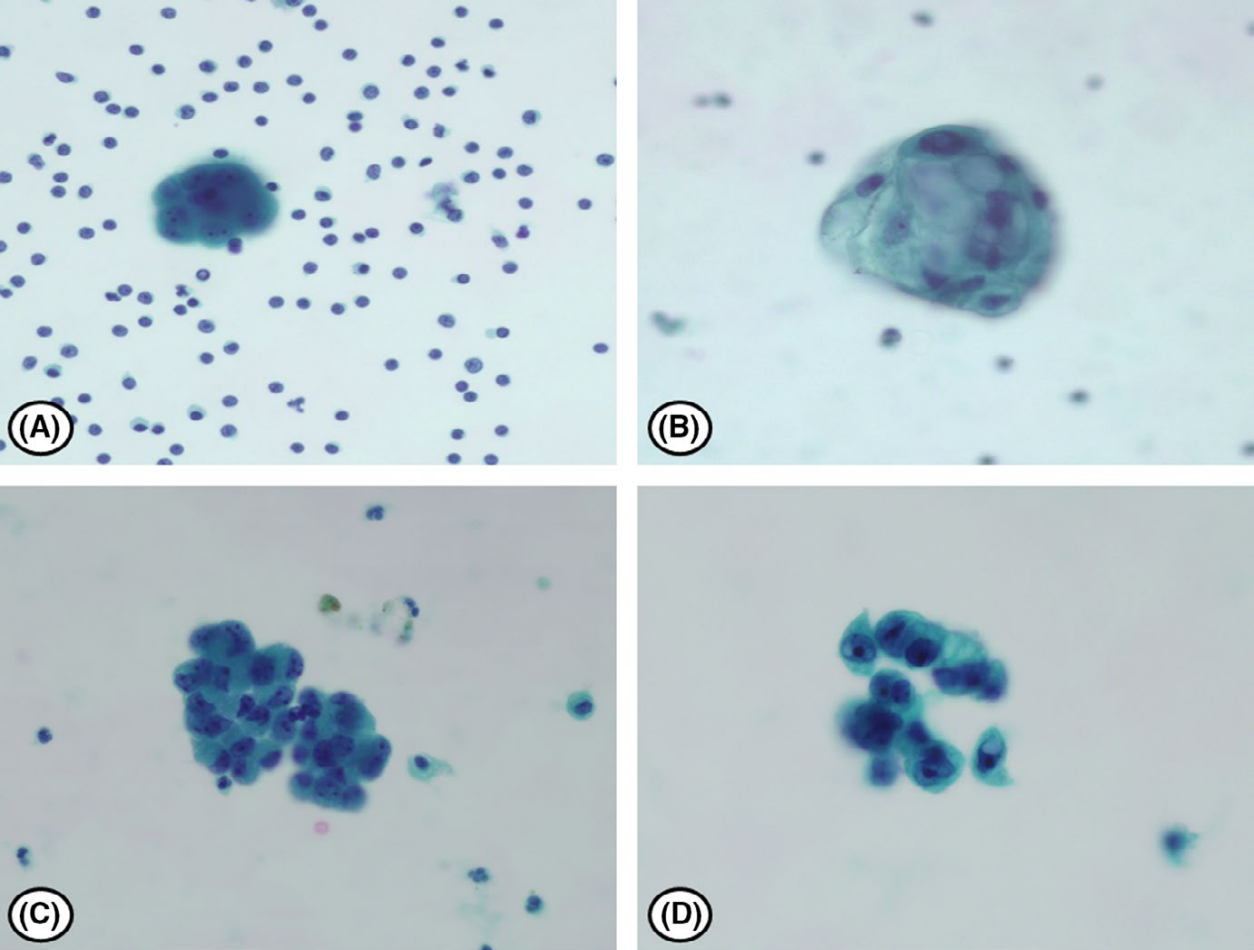
Representative images of negative for malignancy (NFM), atypia of undetermined significance (AUS), and suspicious for malignancy (SFM) in pericardial fluid. (A) NFM shows reactive mesothelial cell and lymphocytes. (B, C) AUS presents reactive mesothelial cells and occasional large atypical cells. A few atypical cells show moderate N/C ratio with hypochromatin. (D) SFM shows atypical cells with high N/C ratio, macronucleoli, and cytoplasmic mucin

**FIGURE 2:**
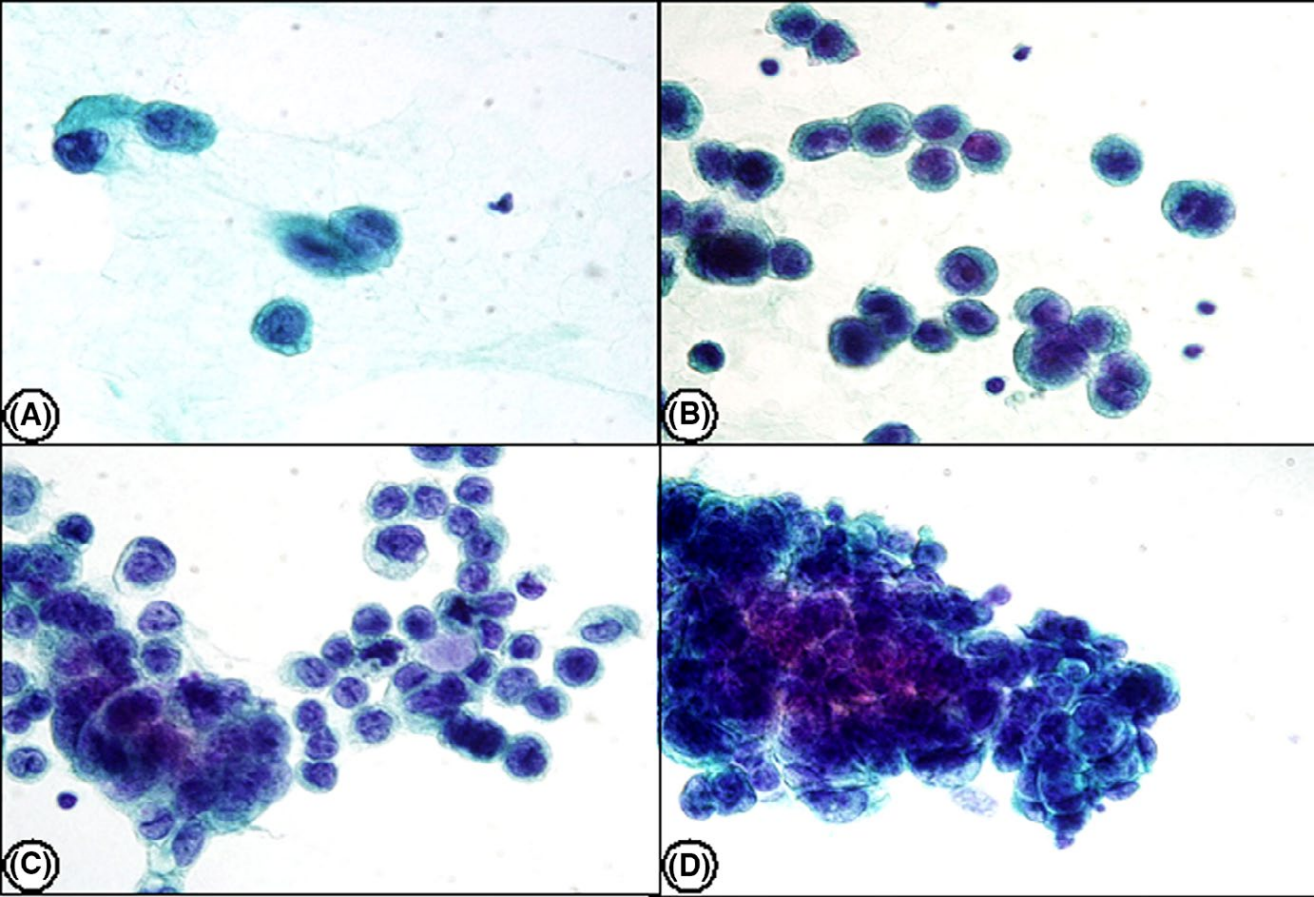
Representative images of malignant pericardial effusion. (A) Adenocarcinoma of the lung, (B) invasive ductal carcinoma of the breast, (C) gastric adenocarcinoma, (D) thymic carcinoma. (Papanicolaou stain, original magnification, ×400)

The cytologic diagnosis of PE was classified into five categories in accordance with ISRSFC.^10^ These categories are non‐diagnostic (ND), negative for malignancy (NFM), atypia of undetermined significance (AUS), suspicious for malignancy (SFM), and positive for malignant cells (MAL). The criteria used for these designation were as follows; (i) ND, extremely scant specimens with no cells or rare benign‐appearing cells (usually less than 10 cells) such as a few macrophages, lymphocytes, mesothelial cells, or RBCs; (ii) NFM, the appearance of one or more type of benign‐appearing mesothelial cells, lymphocytes, blood, and macrophages (iii) AUS, is the assigned categories when (1) there are a few atypical cells of undetermined origin, or (2) atypical lymphocytes, or (3) there are atypical mesothelial cells. (iv) SFM, this category is used when there are (1) recognizable cell types of markedly atypical epithelial cells; (2) markedly atypical lymphocytes, (3) markedly atypical mesothelial cells, or (4) any markedly atypical cells that lead to a suspicion of a specific type of malignancy, such as melanoma. However, there must be insufficient malignant cells for further characterization of malignancy through ancillary studies such as immunohistochemistry (IHC) or flow cytometry to designate the specimen as SFM; (v) MAL, primary and secondary, includes any type of malignancy. Although it is very difficult to diagnose the specific subtype of malignancy using a cytology specimen only, cyto‐morphology characteristics such as increased cell size, increased nuclear‐to‐cytoplasmic ratio, irregular nuclear contours, prominent nucleoli, or coarsely textured chromatin will enable a diagnosis of malignancy. Representative images of NFM, AUS, SFM, and MAL are provided in Figures 1 and 2.

### 2.4 Statistical analysis

A one way ANOVA test was used to analyze variance. Overall survival (OS) was assessed as the interval between the pathologically confirmed date of diagnosis and the date of death from any cause or of the last follow‐up. Survival curves were calculated using the Kaplan–Meier method, and the OS values were compared using the log‐rank test. All reported *p* values were two‐tailed, and those <0.05 were considered statistically significant. Statistical analysis was carried out using IBM SPSS Statistics for Windows, version 21 (IBM Corp.).

## 3 RESULTS

### 3.1 Demographics

A total of 38,589 pleural, peritoneal, and pericardial effusion specimens were processed from 2010 to 2014 in our cytopathology laboratory, from which 574 PE specimens (1.5%, 574/38,589) from 486 patients were analyzed in our current study. The patients consisted of 263 men (54.1%) and 223 women (45.9%) with a median age of 58 years (range, 17–93 years).

### 3.2 Characteristics of the pericardial cytology

The cytologic diagnoses among the 574 PE specimens examined in our study included negative for malignancy (*n* = 382, 66.6%), atypical cells, favor reactive (*n* = 34, 5.9%), atypical cells, suspicious for malignancy (*n* = 10, 1.7%), and positive for malignancy (*n* = 128, 22.3%). These data are summarized in Table 1.

**TABLE 1:** Cytologic diagnosis of pericardial effusion

Diagnosis	Total no. of cases (%)
Negative for malignancy (NFM)	382 (66.6)
Atypia of undetermined significance (AUS)	54 (9.4)
Atypical cells, favor reactive	34 (5.9)
Atypical cells, unknown significance	20 (3.5)
Suspicious for malignancy (SFM)	10 (1.7)
Positive for malignancy (MAL)	128 (22.3)
Total	574

The etiology of the “atypical cells” category was analyzed based on the subsequent specimens or patient's medical reports. These are summarized in Table 2. In brief, the most common etiology for the “atypical cells, favor reactive” was a tumor (*n* = 18, 53%) and followed by heart disease‐related PE (*n* = 3, 8.7%). The most common cause of the “AUS” category was also a tumor (*n* = 8, 40%) followed by hematologic malignancy (*n* = 3, 15%).

**TABLE 2:** Etiology of 54 cases of atypia of undetermined significance (AUS) pericardial effusion without malignancy

Etiology	Total no. of cases (%)
Atypical cells, favor reactive	34 (62.9)
Neoplasm	18 (53)
Hematolymphoid malignancy	2 (5.8)
Heart disease	3 (8.7)
Heart failure	1 (2.9)
Valvular disease	1 (2.9)
Atrial septal defect	1 (2.9)
Tb pericarditis	2 (5.8)
Pericarditis, unknown etiology	1 (2.9)
Renal disease	2 (5.8)
Acute cellular rejection	1 (2.9)
Hypertensive nephrosclerosis	1 (2.9)
Liver cirrhosis	1 (2.9)
EBV‐associated lymphadenopathy	1 (2.9)
Idiopathic	4 (11.6)
Atypical cells, unknown significance	20 (37.1)
Neoplasm	8 (40)
Hematolymphoid malignancy	3 (15)
Amyloidosis	1 (5)
Tb pericarditis	1 (5)
Myocarditis, unknown etiology	1 (5)
Liver cirrhosis	1 (5)
Idiopathic	5 (25)

The most common origin of a malignant PE was the lung (*n* = 86, 82.1%), both in the men (*n* = 45, 70.5%) and women (*n* = 41, 50.6%). The most common subtype of lung cancer was adenocarcinoma in both genders. Breast cancer (*n* = 20, 24.7%) in women and gastric cancer (*n* = 3, 4.9%) in men were the second most frequent cause of malignant PE, respectively. These findings are summarized in Table 3.

**TABLE 3:** Distribution of the primary diagnoses among the cases suspicious for malignancy and positive for malignant pericardial effusion

Primary site	Male (*n* = 59, %)	Female (*n* = 81, %)
Lung	45 (72.0)	41 (50.6)
Adenocarcinoma	37 (59.2)	41 (50.6)
Squamous cell carcinoma	1 (1.6)	0
Adenosquamous carcinoma	1 (1.6)	0
Non‐small cell carcinoma, NOS	2 (3.2)	0
Sarcomatoid carcinoma	1 (1.6)	0
Small cell carcinoma	1 (1.6)	0
Combined small cell and non‐small cell carcinoma	1 (1.6)	0
Plasmacytoma	1 (1.6)	0
Breast	0	20 (24.7)
Mediastinum	5 (8.0)	2 (2.5)
Thymic carcinoma	2	2
Thymoma	1 (1.6)	0
Germ cell tumor	1 (1.6)	0
T‐lymphoblastic lymphoma	1 (1.6)	0
Stomach, adenocarcinoma	3 (4.9)	3 (3.7)
Large intestine, adenocarcinoma	0	4 (4.9)
Female genital tract	0	5 (6.2)
Tonsil, non‐keratinizing carcinoma	1 (1.6)	0
Hepatocellular carcinoma	1 (1.6)	0
Gallbladder adenocarcinoma	1 (1.6)	0
Common bile duct, adenocarcinoma	0	1 (1.2)
Acute myeloid leukemia	1 (1.6)	0
Diffuse large B‐cell lymphoma	0	2 (2.5)
Adenocarcinoma of Unknown primary tumor	2 (3.2)	3 (3.7)

Cell blocks were constructed in 155 cases (27%) and the concordance rate of diagnosis between those of cell blocks and those from the cytology was 78.1%. A pericardial biopsy was performed in 31 (5.4%) out of 574 patient and 9 (29.0%) out of 31 patients were diagnosed as malignancy on pericardial biopsy. Thirteen (41.9%) out of these 31 cases with a pericardial biopsy were classified as SFM or MAL on cytology, but 7 (53.8%) out of 13 cases were simultaneously diagnosed with a malignancy on pericardial biopsy. The sensitivity of pericardial biopsy for the diagnosis of malignancy was 77.8% and the specificity was 72.7% (data not shown).

### 3.3 Outcomes

Follow‐up data of all the cases were obtained in 412 cases which were re‐aspiration specimens after 1st cytologic diagnosis and biopsy of the site of malignancy. Out of these 412 cases, we recategorized in benign and malignant groups. Among the follow‐up data, diagnosed as NFM or AUS specimens were included in a benign group, and diagnosed as SFM or MAL specimens were included in a malignant group. The follow‐up data were used for the calculation of the risk of malignancy (ROM) for each category. Table 4 presents the total number of cases in each category, number of cell blocks, total number of follow‐up, recategorization of follow‐up data, and the calculated ROMs for each category. In the NFM category 382 cases were included, and 268 cases had follow‐up data composed of cytologic specimens. Out of these 268 cases, 10 were malignant, and 120 were benign. The ROM for NFM category was calculated as 3.7%. Among 54 cases of the AUS category were followed for 43 cases. These follow‐up cases were obtained by 18 biopsy specimen and 25 cytologic specimens that contained nine malignant and 34 benign diagnosed. The ROM for this category was calculated as 20.9%. For the SFM category, 10 cases had seven follow‐up data which comprised of two biopsy specimens and five cytologic specimens. After recategorization, four cases were malignant and three cases were benign. The ROM for SFM category was calculated as 57.1%. The MAL category included 128 cases, and 94 cases were followed by biopsy specimens (*n* = 11) and cytologic specimen (*n* = 83). The follow‐up cases were recategorized as malignant (*n* = 84) and benign (*n* = 10). The ROM for this category was calculated as 89.3%.

**TABLE 4:** Cytodiagnosis according to the international system for reporting serous fluid cytopathology (ISRSFC), follow‐up data and calculated ROMs

ISRFSC categories	*n* (%)	Presence of cell blocks (*n*)	Total number of follow‐up (*n*)	Diagnosis on follow‐up (*n*)	Risk of malignancy: no. of malignant cases/no. of cases with follow‐up (%)
Negative for malignancy (NFM)	382 (66.6)	95	268	Malignant (10)	10/268 (3.7%)
				Benign (258)	
Atypia of undetermined significance (AUS)	54 (9.4)	14	43	Malignant (9)	9/43 (20.9%)
				Benign (34)	
Suspicious for malignancy (SFM)	10 (1.7)	7	7	Malignant (4)	4/7 (57.1%)
				Benign (3)	
Positive for malignant cells (MAL)	128 (22.3)	39	94	Malignant (84)	84/94 (89.3%)
				Benign (10)	
Total	574 (100)	155	412	Malignant (107)	107/412 (25.9%)
				Benign (305)	

**FIGURE 3:**
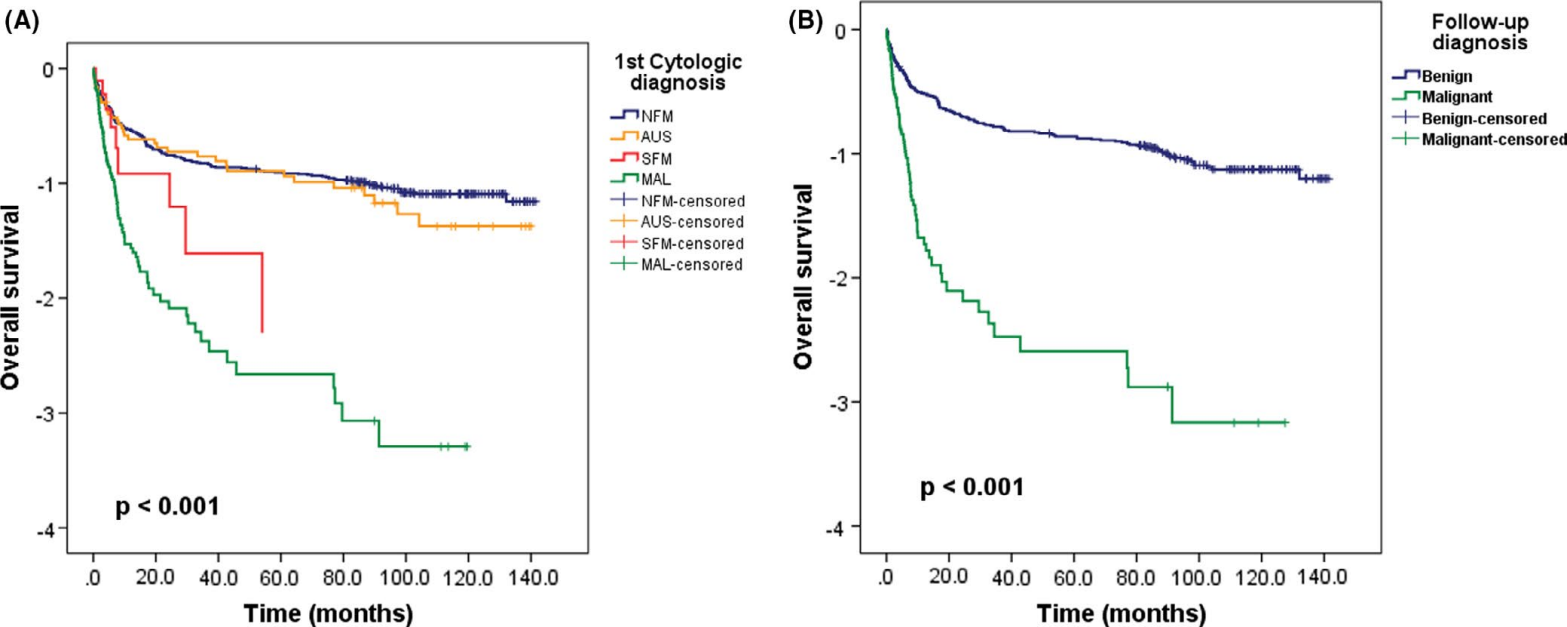
Overall survival (OS) analysis according to an initial diagnosis and follow‐up data. (A) The presence of malignant cells in pericardial effusion showed significant differences in OS compared to absence of malignant cells (*p* < 0.001). (B) The follow‐up data were recategorized in benign and malignant group. The malignant group had poor prognosis than the benign group (*p* < 0.001)

The first diagnosis was classified as four groups and analyzed overall survival (OS). In OS analysis, there were significant differences according to each category of 1st cytologic diagnosis (*p* < 0.001, log‐rank, Figure 3A). MAL had poorer prognosis than SFM, and NFM has better clinical outcomes compared with AUS. Using recategorization follow‐up data for OS analysis, benign group had significantly better prognosis than malignant group (*p* < 0.001, log‐rank, Figure 3B).

Follow‐up data were retrieved for 132 out of 138 patients diagnosed with either “atypical cells, suspicious for malignancy” or “positive for malignancy” to analyze the clinical outcomes in these cases. The mean follow‐up period was 39.1 months (range, 0–222.1 months; median, 18.7 months), and the mean time interval from the date of the initial diagnosis of malignancy to the date of malignant PE occurrence was 29.05 months (range, 0–220.1 months; median, 10.26 months). The mean time interval from the date of occurrence of malignant PE to the date of death was 10.06 months (range, 0–116.03 months; median, 3.5 months), and the 1‐year survival rate was 16.7%. An initial diagnosis of cancer due to detection of malignant PE was made in 24 cases (18.2%), nine (6.8%) of whom died immediately after the onset of malignant PE. Among the cases of malignant PE at the time of this initial diagnosis, 17 (70. 8%) were lung cancer patients, followed by hematologic malignancy in four cases. Lung cancer was the most common cause of the deaths occurring at the same time as the development of malignant PE, followed by hematologic malignancy.

**FIGURE 4:**
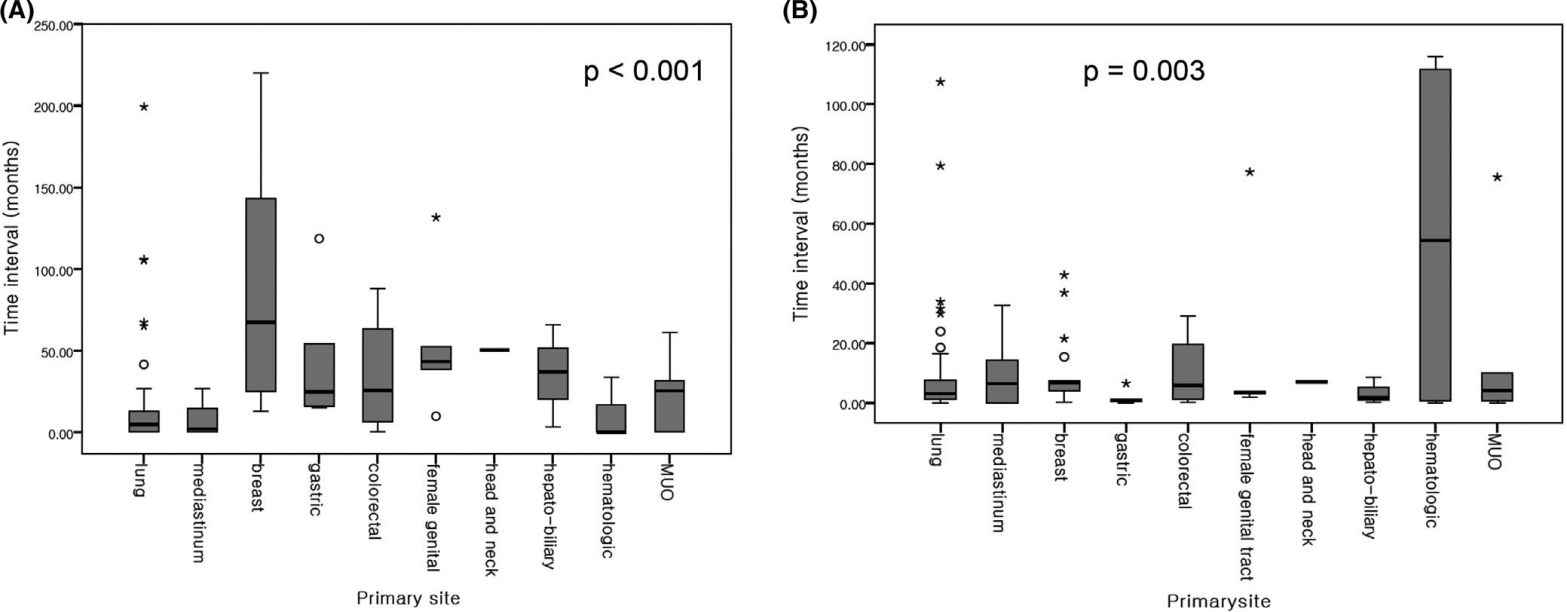
(A) Boxplot of the time interval between the initial diagnosis and first occurrence of malignant pericardial effusion according to the primary site. (B) Boxplot of the time interval between the first diagnosis of a malignant pericardial effusion and the last follow‐up date according to the primary site

The time to onset of the malignant PE after initial diagnosis of malignancy was analyzed according to the type of malignancy (Figure 4). Breast cancer showed the longest duration before the onset of malignant PE with an average of 89.7 months, with the lung and mediastinum showing this onset in a relatively short time, with an average of 15.6 and 8.65 months, respectively. In addition, gastric cancer had the shortest time to death after the occurrence of malignant PE (1.6 months), followed by hepato‐biliary cancer (3.6 months). The corresponding periods for the lung cancer and breast cancer patients were 7.7 and 10.4 months, respectively.

**FIGURE 5:**
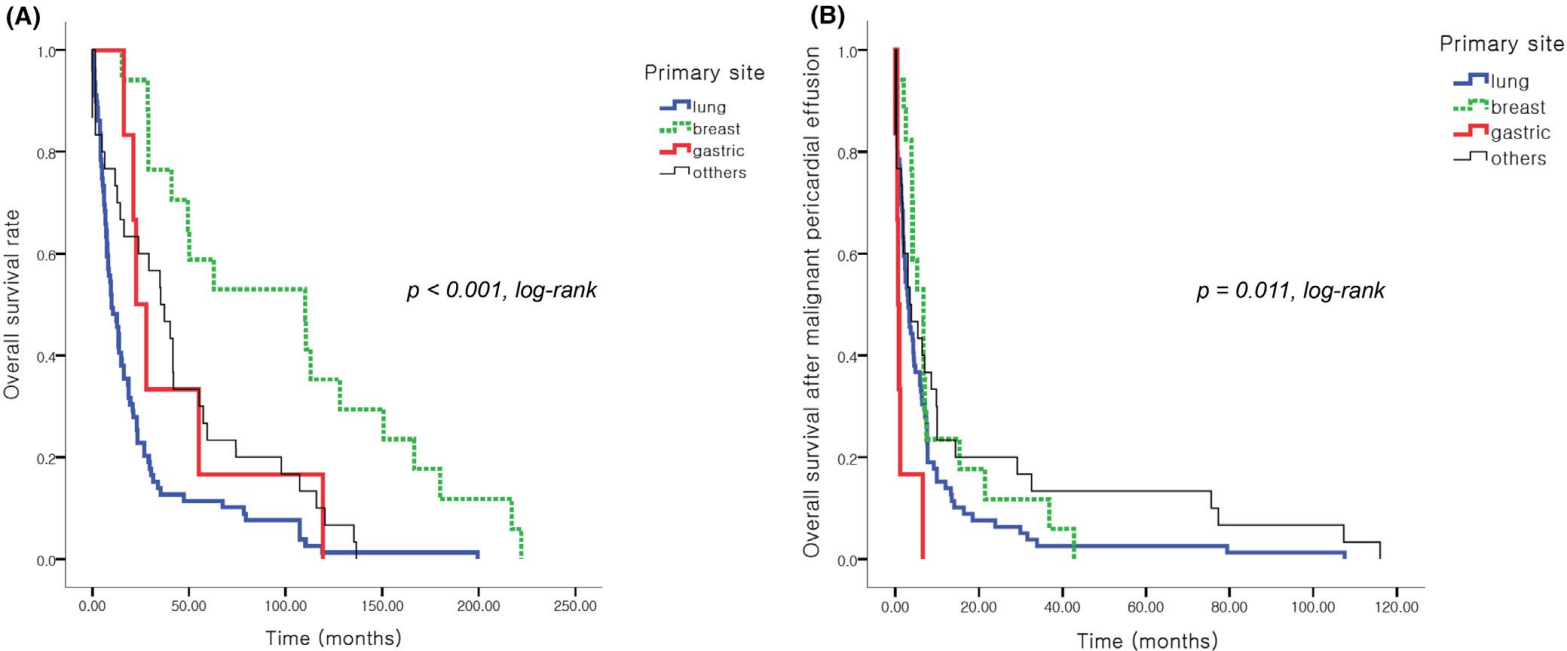
Kaplan–Meier survival analysis. (A) Overall survival rate according to the primary site. Lung cancer shows a poorer prognosis compared to the breast cancer cases (*p* < 0.001, log‐rank test). The specimens include lung cancer (*n* = 79), breast cancer (*n* = 17), gastric cancer (*n* = 6), and others (*n* = 30). The “others” include colorectal cancer, hepato‐biliary cancer, tonsillar cancer, hematologic malignancy, female genital tract, and metastasis of unknown origin. (B) Survival rate according to the primary site after the occurrence of a malignant pericardial effusion

The overall survival rate according to the primary site in the patients with malignant PE revealed that the breast cancer had the best prognosis, and the patients with lung cancer had the poorest prognosis *(p* < 0.001, log‐rank, Figure 5A). Interestingly however, the survival rates after the onset of malignant PE revealed that gastric cancer showed the worst prognosis compared to the others. The 1‐year survival rates after onset of malignant PE were 0% for gastric cancer, 13.9% for lung cancer, 19.8% for breast cancer, and 21.1% for the others (*p* = 0.011, log‐rank, Figure 5B).

## 4 DISCUSSION

We here classified 574 cases, that had all been previously diagnosed with pericardial fluid at our tertiary institute from 2010 to 2014, in accordance with the recently ISRSFC,^10^ and also collected and analyzed the long‐term follow‐up data for these patients. In brief, malignant PE accounted for 24% of all the instances of PE and lung cancer was the most common malignancy to produce malignant PE in both men and women in our study series. After the occurrence of malignant PE, the prognoses differed depending on the type of primary cancer but were all the generally poor, with an average survival time of only 10.6 months.

Prior large‐scale studies involving PE cytology were reviewed^3^, ^9^, ^14^, ^15^, ^16^, ^17^, ^18^, ^19^, ^20^ and are summarized in Table 5. In brief, PE has an incidence of 1.5% to 4.5% among the total body fluids including pleural, pericardial, and peritoneal fluids. The mean age of patients included in those studies were 55.3 years, and the male to female ratio was 1:1. With the exception of previous reports that focused only on malignant PE, the incidence of malignant PE across the studies ranged from 11.3% to 29.5%. The AUS was 1.6%–9.6%. The most common cause of malignant PE was lung cancer in 35.5% to 69.4% of the cases, more prominently in men, and breast cancer was the most common cause in women, accounting for 10.2%–29.0%. There were few data on survival in most of these prior studies. According to a few reports,^15^, ^16^ patients with malignant PE have a survival duration ranging from 5.4 to 10.6 months. Interestingly, the survival times of patients who were diagnosed after 2010 tended to be better than the cases that arose prior to 2000.

**TABLE 5:** Summary of previous published large pericardial effusion cytology series.

Study	Periods	No. of PE pts	Age (mean, years)	Sex	No. of Mal‐PE (MAL/SFM)	AUS	Common etiology of Mal‐PE	Outcomes
García‐Riego et al.	1976–1999	375 (1.6%^a^, total BF *n* = 23,592)	53.6^b^	47 M/18 F	65, 17.4% (65/NA)	NA	Lung (*n* = 38, 58.5%, 33 M/5 F) Breast (*n* = 10, 15.4%, 1 M/9 F)	Median survival^c^: 5.4 mon
Malamou‐Mits et al.	NA (7 years, before 1994)	44	56.9	21 M/23 F	13, 29.5% (13/3)	0 (0%)	Adenocarcinoma (*n* = 6, 46.1%)	NA
Jeong et al.	1991–2010	113	NA	NA	113, 100%	NA	Lung (*n* = 68, 61.9%) Breast (*n* = 17, 15.0%)	Overall median survival: 34.8 mon
Dermawan et al.	2000–2016	1285 (4%^a^, total BF *n* = 30,085)	NA	627 M/658 F	155, 12.1% (15/NA)	58 (4.5%)^d^	Lung (*n* = 55, 35.5%, 28 M/27 F) Breast (*n* = 17, 10.9%)	NA
Kim et al.	2001–2007	98	52	49 M/49 F	98, 100% (98/NA)	NA	Lung (*n* = 68, 69.4%, 38 M/30 F) Breast (*n* = 10, 10.2%)	Mean survival^c^: 7.47 mon 1‐year survival rate: 26%
Dragoescu et al.	2005–2010	128 (4.5%^a^, total BF *n* = 28,16)	52.6	56 M/57 F	31, 24.2% (31/NA)	2 (1.6%)	Lung (*n* = 18, 58.1%, 6 M/12 F) Breast (*n* = 9, 29.0%)	NA
Saab et al.	2008–2014	419	63.0	176 M/188 F	62, 15% (62/NA)	25 (6%)^d^	Lung (*n* = 9, 47.4%, 9 M) Lung and breast (*n* = 11, 39.3%, 11 F)	NA
Rodriguez et al.	2014–2019	299	51.25	163 M/136 F	34, 11.3% (30/4)	13 (4.3%)	Lung (*n* = 11, 36.7%) Breast (*n* = 7, 23.3%)	NA
Present study	2010–2014	574 (1.5%^a^, total BF *n* = 38,589)	58.0	263 M/223 F	138, 24.0% (128/10)	54 (9.6%)	Lung (*n* = 96, 62.3%, 45 M/41 F) Breast (*n* = 20, 14.5%)	Mean survival^c^: 10.6 mon 1‐year survival rate: 16.7%

Abbreviations: AUS, atypia of unknown significance; BF, body fluid; F, female; M, male; MAL, malignancy; Mal‐PE, malignant pericardial effusion; mon, months; NA, not available; PE, pericardial effusion; Pts, patients; SFM, suspicious for malignancy.

^a^ Number of pericardial effusion cases among those for total body fluids including pleural, peritoneal, and pericardial effusions.

^b^ Number of patients with malignant pericardial effusion.

^c^ This survival rate indicates the time from the onset of pericardial effusion to death.

^d^ This includes “atypical cells present or suspicious for malignancy”.

Dragoescu et al.^9^ have proposed that the greater rarity of PE, and the fact that the effusion in these cases is usually not tapped until the onset of the cardiac tamponade, underlies why there have been very few studies on it compared to pleural or peritoneal cytology. Saab et al. have reported that the average pericardial fluid volume measures from 49 to 83 ml, according to the cytologic diagnosis.^18^ Moreover, when pleural and pericardial effusions occur simultaneously, only the pleural effusion tends to be drawn. Hence, the collection of a large number of PF cytology cases for a systematic analysis is difficult to accomplish.

According to the ISRSFC,^10^ the ROM by each category is 17.4 ± 8.9% for ND, 21 ± 0.3% for NFM, 66 ± 10.6% for AUS, 82 ± 4.8% for SFM, and 99 ± 0.1% for MAL. Compared with our results, ROM of MAL was approximate to the value of ISRSFC but ROM of SFM was low than the value of ISRSFC. On the other hand, our study estimated low ROM in NFM cases. The diagnostic criteria for each category were applied to our cases properly and also we concerned not only cytologic feature, but also background conditions such as inflammatory cells, mesothelial cell proliferation, or necrotic debris. This was helpful to diagnose appropriately and would make less mismanagement.

AUS are defined as a specimen that lacks the quantitative or qualitative cytologic features that can be confidently diagnosed as either benign or malignant and that exhibits sufficiently clear morphologic features to exclude the possibility of classifying them as ND. Our study analyzed AUS as two categories, “atypical cells, favor reactive” and “atypical cells, unknown significance.” “Atypical cells, favor reactive” included cells with a low risk of the malignancy, with reactive macrophage, and mesothelial cells. “Atypical cells, unknown significance” included cells of an uncertain nature, particularly degenerated bland looking tumor cells due to poor preservation. Interestingly, our study indicated that the common cause of these categories was a neoplasm, accounting for 53% and 40%, respectively. The ISRSFC system indicates an expected incidence of an AUS category in the pleural fluid of between 0.6% and 1.6%^21^ and that the incidence of AUS in PE cases would be increased. However, the incidence of AUS has been reported from 1.6% to 9.6%^9^, ^14^, ^17^, ^18^ including our present study. Our study also found a relatively high incidence of AUS due to our categorization of “atypical cells, favor reactive”. If the category of “atypical cells, favor reactive” excluded, the rate of “atypical cells, unknown significance” was 3.5%, which was similar to previous studies.^14^, ^17^

Dragoescu et al.^9^ have suggested previously that the pericardial cytology is better than a pericardial biopsy for detecting a malignancy with a sensitivity of 71% and a specificity of 100% compared to 64% and 85%, respectively. We observed a similar sensitivity and specificity for malignancy detection using pericardial biopsy.

Regardless of gender, the most common primary lesion for a malignant PE was found to be the lung, consistent with almost all previous studies.^3^, ^9^, ^14^, ^15^, ^17^, ^18^ In the case of women, there was a slight difference according to the literature, lung cancer was still the most common primary lesion, followed by breast cancer. Among the extra‐thoracic origin tumors, stomach cancer was the most common in the men and malignancy of the female genital tract origin was most prominent in the women, which were similar results to those reported in previous study.^14^

In our study, only patients with PE were studied, which was a limitation in terms of fully understanding the prognosis in accordance with the course of the malignant tumors. Jeong et al.^19^ revealed that the prognosis was worsen in cancer patients with malignant PE compared to patients without malignant PE (*p* = 0.002).

Prior studies on the clinical outcomes of malignant PE have reported a 1‐year survival rate range of 10%–27%^15^, ^20^, ^22^, ^23^ and a mean survival after malignant PE ranging from 5.4 to 8 months.^15^, ^20^, ^22^, ^23^ Interestingly, our study revealed for the first time that the prognosis of patients after the occurrence of malignant PE was generally poor, but that gastric cancer patients had the worst outcomes. In addition, our study showed slightly increased survival time compared to previous studies. When considering the possible reasons for this, it must first, be noted that since the prognosis for breast cancer is generally better than that of other carcinomas, it can be assumed that the overall survival time will increase if there are more breast cancer cases in the included patient group. Second, the years of diagnosis among the study patients are important as targeted therapy has been conducted since 2000, especially in breast and lung cancer, it can be seen that the period of inclusion of the patient group affects the prognosis. A large‐scale study with a more recent study population is thus needed.

In conclusion, the current study is the first to our knowledge to classify the pericardial fluid in 574 PE according to the recently published ISRSFC guidelines and to present the long‐term outcomes of the patients with malignant PE at the same time. This study revealed significant differences in prognosis according to each category of ISRSFC. Also, analysis of follow‐up data supported a better overall survival tendency toward benign group than malignant group. The majority of follow‐up data were composed of cytologic specimens and evaluated. The results of follow‐up data correlated with clinical outcome showed that a cytologic evaluation is useful than a pericardial biopsy. Of particular note, the gastric cancer patients in our study showed the poorest prognosis after the occurrence of malignant PE, which differs from previous reports. In addition, we found that a cytologic evaluation of PE is more useful, minimally invasive method for diagnosing metastatic carcinoma than a pericardial biopsy.

## ETHICS APPROVAL AND CONSENT TO PARTICIPATE

The use of patient material in this study was approved by the Institutional Review Board of Asan Medical Center, Republic of Korea (2021‐0878).

## CONFLICT OF INTEREST

The authors declare no competing interest.

## Data Availability

Not applicable. Data sharing is not applicable for this article as no datasets were generated or analyzed during the current study.
